# Temperature Variations in Pulmonary Tuberculosis

**Published:** 1907-11-23

**Authors:** H. Hyslop Thomson

**Affiliations:** formerly Medical Superintendent, Consumption Sanatorium, Bridge of Weir, N.B.


### SPECIAL ARTICLE.

### TEMPERATURE VARIATIONS IN PULMONARY TUBERCULOSIS.

By H. HYSLOP THOMSON, M.D., formerly Medical Superintendent, Consumption Sanatorium, Bridge of Weir, N.B.

The clinical study of pulmonary tuberculosis presents to the physician many features of great interest. When the disease has encroached to any considerable extent on the aërating pulmonary surface, or has displayed an acute and progressive character, there is nearly always a well-marked deviation from the normal in the temperature of the body. The presence of fever is common in many acute microbic diseases, but in no morbid condition is the type of pyrexia so variable as in pulmonary tuberculosis. It is generally accepted that the amount of fever present in any case of pulmonary tuberculosis may be regarded as a fairly accurate gauge of the intensity of the infective process in the pulmonary tissue. The more acute and rapidly progressive the infective process the higher is the temperature. Moreover, the extent and type of the fever appear to stand in somewhat close relation to the amount and character of toxin absorbed into the systemic circulation. At the same time it must not be overlooked that tuberculosis of the lungs does occasionally occur in an acute form without any well-marked pyrexia, and further, that in some cases the mean temperature remains persistently below normal, notwithstanding progressive activity in the pulmonary lesion.

We may accept as a working basis that in the majority of cases the fever characteristic of pulmonary tuberculosis is due to absorption into the systemic circulation of toxic products of local bacterial growth. With the specific micro-organism, pyogenic cocci and occasionally non-pathogenic saprophytic bacteria are frequently associated. Occasionally extra-pulmonary etiological factors are at work.

This condition of toxæmia throws out of balance the nerve mechanism concerned with thermal production and dissipation; but in some cases the micro-organisms themselves may find their way into the blood-stream and lead to the development of generalised miliary tuberculosis, or give rise to the classical symptoms of a septicæmia.

The exact nature of the toxic agents responsible for the fever of tuberculosis is still the cause of some diversity of opinion. Koch, to whose opinion due consideration must be given, has stated that if the temperature in pulmonary tuberculosis rises much above 100° F., this is to be regarded as strongly in favour of mixed infection, and that cases of pure tuberculosis remain more or less afebrile throughout. In a recent article Galbraith [1] voices the same opinion, and many other competent observers appear to hold similar views. But from the clinical standpoint this view regarding the non-febrile effect of the tubercle toxin is untenable.

In the first place, there is the definite febrile reaction called forth by the injection of tuberculin. The subcutaneous injection of 1 milligramme of T.R. into the subject of quiescent tuberculosis produces a two-fold reaction. There is a local reaction around the quiescent focus in the lung itself, evidenced by increased cough and sputum, quickened breathing, and occasional streaky hæmoptysis. Associated with local we have evidence of constitutional disturbance, in the quickened pulse, elevation of temperature, and general malaise. The most striking feature of the disturbance is its similarity to the clinical picture of sudden severe recrudescence in a quiescent chronic case. An estimate of the opsonic index in the case of a patient with quiescent tubercle who has received an injection of tuberculin, reveals a well-marked negative phase, and it is a reasonable supposition that during this fall in the resisting powers of the tissues a certain amount of toxic absorption occurs which finds expression in the general disturbance already referred to.

The contention that the toxin of tubercle is responsible itself for pyrexia gains support from the high continued fever often seen in miliary tuberculosis. The suggestion that in miliary tuberculosis of the lungs there is not a pure infection by tubercle but a mixed infection, and that the activity of certain pyogenic bacteria must be held accountable for the high fever met with in this condition is negatived alike by the symptoms and course of the disease and by the post-mortem findings. It therefore seems beside the point altogether to attribute the fever of miliary tuberculosis to any toxins other than those of the tubercle bacillus.

In addition to miliary tuberculosis there are certain other forms of closed tuberculosis which are frequently associated with a high range of fever. Primary acute tuberculous pleurisy is usually ushered in with a well-marked access of fever.

In closed surgical tuberculosis also where the disease is completely shut off from the possibility of septic contamination we not infrequently find considerable elevations of temperature. I am indebted to Dr. Dalziel of Glasgow for particulars regarding the following cases. The first was a child who, for many weeks, had suffered from tuberculous peritonitis with high fever. The abdomen was opened and, on bacteriological examination, a pure culture of tubercle bacilli was obtained. Following the operation there was marked improvement in the patient's condition and the temperature fell to within normal limits. The second was a young woman with large masses of tuberculous glands in the neck. The glands were hard and showed no evidence of breaking down. For some weeks there had been high fever, the maximum reached being 104° F. The glands were removed, subsequent to which the temperature fell to normal, and the patient made an excellent recovery. In a case of

[1] "Practitioner," February 1906.

tuberculous lymphadenitis mentioned by Osler,[2] the chart revealed persistent high fever. Apart from one or two puckered spots at the apex, the post-mortem examination revealed no disease except tuberculosis of the cervical, bronchial, retroperitoneal and mesenteric glands. These examples show that the toxins of tubercle are capable of producing high grades of fever.

When a tuberculous lesion becomes open there is great risk of septic infection supervening; the characteristic fever of which shows well-marked excursions. In such cases the combined toxic products of the tubercle bacilli and of certain pyogenic bacteria are absorbed, but whether such products are antagonistic or complemental has yet to be proved. Clinically we know that the association of active pyogenic micro-organisms with tubercle bacilli in the same lesion is responsible for many of the distressing symptoms seen in advanced pulmonary tuberculosis. The amount of toxæmia in pulmonary tuberculosis varies within wide limits, even in the same individual. It depends upon the virulence of the infective bacteria, and the natural resistance offered by the tissues to the development of the morbid process. Broadly, the amount of systemic infection increases *pro rata* with the morbid process. But even in what are clinically recognised as non-acute cases we have the entrance of relays of small quantities of toxin into the circulation, and this favours the subsequent development of acute symptoms. Wright has shown that the resistance of the tissues to certain infective processes may be gauged by the amount of a (hypothetical) opsonin in the blood serum. The presence of a slight degree of toxæmia may lead to a diminution of opsonin in the blood and concurrently to a diminished capacity of the cells to deal with infective bacteria; in such an event the disease becomes more active, and further elaboration and absorption of toxin take place.

A mean subnormal temperature in pulmonary tuberculosis is usually met with in one of three distinct pathological conditions. (1) Early benign cases of asthenic type; (2) chronic benign cases with well marked fibrosis; and (3) advanced cases with marked asthenia and evidence of failing heart.

The causation of subnormal temperature in these conditions offers an interesting subject for consideration. It has been suggested that the tubercle bacillus is capable of producing a toxin which interferes with normal heat production, and no doubt this is the true etiological factor in the subnormal temperature of certain cases. Indeed, the various clinical manifestations of closed tuberculosis can only be explained on the ground that the tubercle bacillus elaborates different toxins under varying conditions.

In addition to toxæmia as a factor in the production of fever there are certain extra-pulmonary causes which are directly responsible for well-marked elevations of temperature. These are a septic condition of the mouth, auto-intoxication from the intestinal tract, and the neurasthenic temperament. The importance of oral sepsis as a contributing factor in the fever of pulmonary tuberculosis has been frequently pointed out, and it is noteworthy that a high range of fever sometimes declines if the pyorrhœa alveolaris yields to active antiseptic treatment. The effect of Rigg's disease on the course of pulmonary tuberculosis is a serious one, and its development exercises a very grave influence on both the immediate and ultimate outlooks.

[2] "Principles and Practice of Medicine."

Sudden excursions of fever in cases of chronic and subacute phthisis are not infrequently associated with symptoms pointing to absorption from the intestinal tract. There may be abdominal pain, with diarrhœa or constipation. The tongue is foul, urine high coloured, and headache and general malaise are complained of. The temperature is high, the pulse rapid, and there may be slight icterus. A suggestive sidelight is thrown on this toxic condition by Woods Hutchison[3] in a recent paper on "The Liver as a Toxin Filter." Placed between the portal and systemic circulation, the liver arrests and destroys toxic agents elaborated by intestinal fermentation and putrefaction. When such toxic products are present in excess, or if the liver, owing to some inherent changes in its structure, is incapable of performing its normal function, a toxic overflow into the systemic circulation gives rise to constitutional symptoms. In chronic and subacute pulmonary tuberculosis this auto-toxæmia is prone to occur. In such cases, when under sanatorium treatment, food is taken in considerable quantity and disturbance of the digestive function may ensue, resulting in intestinal trouble. Moreover, the liver in phthisis is frequently less capable of performing its normal function as filter owing to fatty infiltration or amyloid disease. That the fever and other symptoms described are, indeed, due to auto-intoxication from the intestinal tract has been frequently proved by the manner in which they yield to intestinal antisepsis induced by the administration of calomel and salol.

### Conclusions.

1. There are two clinical types of closed tuberculosis of the lungs, one of slow development, the other characterised by extreme virulence.
2. In the slowly developing form the temperature is either subnormal or but slightly elevated in the after part of the day.
3. In the virulent form the temperature is usually high, and has a tendency to approximate the continued type.
4. In open tuberculosis with mixed infection, when the tubercle bacilli and pyogenic bacteria are equally active, remittent fever is usually met with.
5. In cases of cavity formation with predominant septic infection the fever is most frequently of the intermittent type.
6. Exceptionally in progressive phthisis of a somewhat acute type pyrexia is absent, or the mean temperature is persistently subnormal.
7. A subnormal temperature is a characteristic feature in early asthenic cases, and in chronic fibrotic phthisis.
8. Sudden excursions of fever in chronic and subacute phthisis may be due to causes other than toxic absorption from the pulmonary lesions.

[3] Practitioner, November 1906.

